# Activated clotting time in inpatient diagnostic and interventional settings

**DOI:** 10.1007/s11239-022-02672-y

**Published:** 2022-06-22

**Authors:** Heidi Dalton, Michael Martin, Pamela Garcia-Filion, David Shavelle, Pei-Hsiu Huang, Justin Clark, Sean Beinart, Andrew Israel, Nichole Korpi-Steiner

**Affiliations:** 1 Inova Fairfax Hospital, Falls Church, VA USA; 2 San Diego, CA USA; 3 University of Arizona College of Medicine, Phoenix, USA; 4 University of Southern California Medical Center, Los Angeles, CA USA; 5 Sutter Valley Hospital, Sacramento, CA USA; 6 Charleston Area Medical Center, Charleston, WV USA; 7 Washington Adventist Hospital, Takoma Park, MD USA; 8 Robert Wood Johnson Hospital, New Brunswick, NJ USA; 9 University of North Carolina Medical Center, Chapel Hill, NC USA

**Keywords:** Activated clotting time, Cardiac Catheterization, Cardiopulmonary bypass, Extracorporeal life support, GEM Hemochron 100, Hemochron Signature Elite

## Abstract

Monitoring for the anticoagulant effect of unfractionated (UFH) at the point of care using activated clotting time in real time is vital where risk of thrombosis is high. Although monitoring UFH effect is a routine and important task, changing from one ACT instrument type or technology to another must be preceded by a clinical and statistical evaluation to determine the suitability and repeatability and establish normal and treatable ranges of this newer instrument. In this multi-center prospective evaluation we tested 1236 paired ACT+ samples, and 463 paired ACT-LR samples (1699 total) from enrolled study subjects. Clinical settings included CVOR cardiopulmonary bypass, at the beside in extracorporeal life support (ELS), the Cardiac Catheterization Lab (CCL) during diagnostic studies and percutaneous coronary interventions (PCI), interventional radiology procedures and EP interventions. This study found more consistent clinical performance from the GEM Hemochron 100 as compared to the current clinical model, the Hemochron Signature Elite. The bias of GEM Hemochron 100 for ACT+ and ACT-LR was greatest in the setting of the CVOR where ACT levels were high. ACT-LR measurements by the GEM Hemochron 100 were comparable to the SE when performed in settings of CCL, ECM, EP and ICU. Results obtained for both ACT-LR and ACT+ in all clinical settings in this study using the GEM Hemochron 100 are as accurate and more repeatable as those with the current clinically available Signature Elite.

## Introduction

Negative observations have been made by several investigators in their studies where lower than desirable repeatability [1, 2] was observed. The present study looked closely at repeatability in a new ACT instrument as compared to the currently available (predicate) technology.

Many diseases and related treatments use anticoagulant medications to prevent thrombosis. UFH, first discovered in 1916, is one of the oldest and most commonly used medications for anticoagulation. Intravenous administration of unfractionated UFH (UFH) is prescribed for preventing thrombosis and/or limiting the extension of existing clots within the body [3]. UFH is generally used for anticoagulation in conditions such as acute coronary syndrome (ACS), atrial fibrillation, deep-vein thrombosis, pulmonary embolism, and others. UFH is also administered or in other conditions where risk of thrombosis is high such as in the cardiac catheterization lab (CCL), during percutaneous coronary interventions (PCI) and interventional radiology (IVR), during CPB for heart surgery and in extracorporeal life support (ELS) to help prevent clotting in the circuit. Some clinicians believe monitoring the anticoagulation effects of UFH with ACT is advantageous, because ACT represents clotting of whole blood, not just UFH effect or circulating UFH, and it gives the best overall picture of the coagulation status. Well accepted and extensively research published in *Clinical Practice Guidelines-Anticoagulation during Cardiopulmonary Bypass* [4], points out that ACT correlates more closely with factor Xa activity, is less affected by hypothermia and artifacts, allow for shorter testing time, and removes variability induced by hemodilution. Still some clinicians find that the lack of correlation between UFH dose and ACT presents a challenge to them in adjusting UFH dosing.

Monitoring for the anticoagulant effect of UFH is performed by a variety of tests, such as activated clotting time (ACT), activated partial thromboplastin time (aPTT), anti-Xa, or clotting time via viscoelastic testing [5]. Although all these tests have strengths and limitations, the ACT remains the most commonly used test to monitor UFH effect and adjust UFH dose in the acute care setting.

### Objective

The primary objective of this multi-center prospective study was to demonstrate if results obtained from a newer generation of Hemochron instrument, the GEM Hemochron 100 (GEM H100) ACT (Werfen, San Diego, CA) measurements are clinically equivalent and more repeatable to those of the 510(k) cleared predicate device, the Hemochron signature elite (SE) ACT (Werfen, San Diego, CA) in a large and varied cohort. Such testing is an important task prior to introducing a new device in the clinical setting.

### Activated clotting time testing

ACT was first developed in 1966 by Hattersley [3]. However, Bull et al. suggested that Hattersley’s test could be applied to coagulation monitoring of the heparinized cardiopulmonary patient [6].

The ACT is normally performed as a point of care test (POC) using whole blood, which is placed in a tube or cartridge and activated with a pro-coagulant substance to initiate the clotting cascade. While ACT has been used for many years in UFH management at the POC, it is important to remember that it represents clotting of whole blood, not just UFH effect or circulating UFH. Some clinicians believe this is advantageous, as the ACT gives an overall picture of the coagulation status in the body. Others find the lack of correlation between UFH dose and ACT a disadvantage when trying to apply ACT level as the means to adjust UFH dosing for optimal effects. The correlation between ACT measurement and circulating UFH or a specific UFH dose is variable because all patients have a unique response to UFH dosing. ACT is primarily affected by circulating UFH, and therefore the target range of ACT differs based on clinical setting, where UFH dosing varies significantly. However, because ACT is a nonspecific measurement of whole blood coagulation, anything that affects coagulation will ultimately affect the ACT. During CPB this is compounded by other variables which impact ACT such as hemodilution, hypothermia and other factors. Genetic factors may also play a yet undefined role in ACT and coagulation. Stoichiometric differences between lots of UFH may also result in correlation differences between dosing and observed measures, as UFH requires a specific pentasaccharide sequence to bind to antithrombin and exert anticoagulation effects [7–9].

Usual clinical therapeutic ranges of ACT vary from 130 to 600 s, with higher results occurring following a large dose of UFH such as used during CPB.

## Materials

We tested 1236 paired ACT+ (Kaolin and Silica activators) samples, and 463 paired ACT-LR (Celite and Silica activators) samples from enrolled study subjects over a 4-month period. Characteristics of the study cohort across all sites are detailed in Table 1.

**Table 1:** Characteristics of the study cohort

	ACT+ (n = 1236)	ACT-LR (n = 463)
Male	68.8 (850)	65.4 (303)
Age (mean ± SD)	63.1 ± 12.5	65.5 ± 15.6
Ht (cm)	171.6 ± 11.0	169.7 ± 11.1
Wt (kg)	86.1 ± 21.6	84.3 ± 21.5
Setting [n(%)]		
CCL	–	204 (44)
CVOR	1088 (88)	147 (32)
ECMO	–	73 (16)
EP	147 (12)	23 (5)
ICU	–	16 (4)

*CCL* Cardiac Cath Lab; *CVOR* cardiovascular operating room; *ECMO* extracorporeal membrane oxygenation; *EP* electrophysiology; *ICU* intensive care unit

Blood sampling for the study was assessed in lower ranges of ACT (up to 400 s) via the ACT-low range (LR) cartridge (up to < 2.5 units/mL of UFH) for patients receiving extracorporeal life support, UFH therapy in the ICU, diagnostic or interventional cardiac catheterization laboratory (CCL) and invasive Radiology procedures, and in the higher range (up to 1005 s) via the ACT+ cartridge (from 1.0 to 6.0 units/mL of UFH) typically used for patients receiving larger UFH doses during in CPB or electrophysiology (EP) interventions. Table 4 lists the procedures where ACT was tested for this study.

The GEM Hemochron 100 is a new version of the Hemochron SE instrument and incorporates modernized optics, and sample processing hardware and software. Comparison of new technology (GEM Hemochron 100) to the current clinically used instrument (Hemochron SE ACT) is essential in any clinical setting prior to introducing it into clinical practice.

Similar to the predicate SE instrument, the GEM Hemochron 100 incorporates a test chamber within a single use cartridge, which are specified by the manufacturer for either low range (< 2.5 units/mL) or high range (from 1 to 6 units/mL) indications. A fresh whole blood sample of 1 to 2 drops of blood (35 µL) is manually dispensed into the test cartridge and warmed to 37 °C (98.6°F) ± 1 °C. The instrument then draws a precise volume of the whole blood sample into the cartridge containing the reagent required to perform the respective coagulation assay. Blood is mixed with a reagent and actively pumped back and forth across a restriction in the test channel to induce clotting. As clotting begins to occur, the movement of the blood decreases below a pre-determined rate which indicates clot formation. The time in ACT seconds required to reach this endpoint is calculated and the results are displayed on the touch screen and stored in the instrument.

Differences between the Signature Elite and GEM Hemochron 100 instrument consisted of software algorithm and hardware improvements in the GEM Hemochron 100 instrument. These changes were designed to produce more consistent and repeatable ACT measurements.

### Sites and participants

Evaluation of the ACT LR was performed in patients receiving extracorporeal life support (ECLS), and from clinical areas where anticipated ACTs are < 400 s. The vast majority of patients within the interventional cardiology suite or operating theater had ACT+ tests performed because of anticipated ACT > 400 s.

ACT-LR study sites were Inova Health Systems/Fairfax Medical Center, Sutter Health/Sutter Valley Hospital, and University of Southern California Medical Center. ACT+ study sites were University of North Carolina Medical Center, Charleston Area Medical Center and Washington Adventist Hospital. Both ACT-LR and ACT+ samples were collected at Robert Wood Johnson Hospital.

Each site was chosen for their specific representation of different operators, clinical settings and conditions expected during routine clinical use. Participating users were trained in the device operation and all testing was performed at the patient’s point of care. Central clinical laboratory personnel or facilities were not used for this investigation.

## Methods

To eliminate the possibility of individual operator and or clinical setting contribution and undue influence on the data, this investigation was conducted as a prospective convenience sample study at seven clinical sites in the United States.

Samples were placed in their respective ACT test cartridge for the SE and GEM Hemochron 100 devices simultaneously and immediately after being drawn. Tests were performed by trained personnel representative of perfusionists, research assistants, nurses and technologists who run ACT tests in their clinical environment. Along with the time and date of each sample and ACT values from the test instruments, patient data (age, weight, gender, room temperature, hematocrit, and hemoglobin).

### Testing procedure

All ex-vivo fresh whole blood samples were tested simultaneously on 2 investigational GEM 100 instruments and 2 predicate SE instruments with ACT+ or ACT-LR cartridges in clinical settings which included the cardiovascular operating room (CVOR), CCL, IVR, EP lab, during ECLS, and in patients receiving parenteral UFH therapy in the ICU. Incorporation of patients in varying circumstances and at differing clinical sites allowed a distribution of ACT values necessary to evaluate clinical accuracy over the range of expected non heparinized and heparinized patient’s therapeutic values.

Technique and workflow for the ACT testing process was consistent across all sites and was representative of those routinely encountered in elective and emergent clinical environments where ACT is used in the management of UFH dosing. On each day of testing, an external liquid quality control (LQC) procedure per protocol was performed on each of the investigational study instruments to ensure that the device was performing properly. Any instruments failing the quality control process were replaced preemptively if needed prior to data collection for that day.

### Inclusion criteria

- Males and females age 18 years or older.
- Patients requiring anticoagulation with UFH during scheduled for elective or urgent procedures where rapid test results are vital.
- Patients requiring UFH anticoagulant therapy for any approved indication and being managed and monitored with the ACT test as part of routine clinical care.

### Exclusion criteria

- Patient developed a significant adverse event before the first whole blood sample is drawn, or
- Patients with a hematocrit of less than 20% or greater than 55%. These samples are not recommended for testing due to optical densities outside of the instrument levels of detection which is common for ACT instruments.

### Ethical considerations

ACT results generated from the GEM Hemochron 100 instrument were used solely for research purposes and were not used for medical or anticoagulation management of study subjects.

The study sites and Investigators were approved by the Western institutional review board (WIRB) including waiver of written informed consent using leftover samples after other physician ordered routine testing on each subject’s whole blood. This study was conducted in compliance with the protocol, the code of federal regulations (CFR), the international council for harmonization of technical requirements standards (ICH) and good clinical practice (GCP).

### Sample size rationale

Clinical and laboratory standards institute (CLSI) standard EP09-A3i states that at least 100 patient samples across the entire measurement range be collected in order to have sufficient evaluable ACT sample results of each assay type to comply with their guidelines. EP09-A3 also recommends that these values span as much of the common clinical measuring range of both cuvette types (ACT+ and LR) as feasible. In addition, past communication with regulatory authorities has indicated that 50 or more measurements at each of the 3 important decision points (normal, therapeutic, and high) is desirable [10]. Simulations performed by the study statistician indicated that at least 120 data points are required for the multi-site analysis. Therefore, a minimum of 150 evaluable samples were required for each cartridge type in order to apply the pre-determined acceptance criteria for the multi-site data of Passing–Bablok Slope between 0.90 and 1.10, the r ≥ 0.90 and Bias of ≤ 10%.

### Analysis methodology

The analysis methodology for data from individual sites and for all pooled multi-site data, was performed according to the predetermined data analysis plan in the study protocol. This included estimates of differences and correlations between the investigational instruments and the predicate SE instruments. These analyses encompassed linear regressions using Passing–Bablok analysis with intercept and slope of the data (along with the 95% confidence interval associated with each estimate), Bland–Altman plots of the differences, Pearson product-moment correlation coefficient (r), overall average bias and bias at predefined clinically important decision points. These variables were calculated for first sample replicate and averages between the two ACT values from each instrument type at ranges appropriate for the cuvette type.

### Statistical analysis

The data were analyzed using Stata 14.0 (College Station, TX) to determine the agreement and accuracy of the GEM H100, the investigational instrument, compared to the predicate SE (reference). Univariate summary statistics for ACT measurements by instrument are presented as the median (50th %ile) and interquartile (25th %ile, 75th %ile) range (IQR); categorical data are reported as relative frequencies. For bivariate analyses, ACT measurements were stratified as ACT+ (up to 800 s) and ACT-LR (up to 400 s) and were compared to assess the repeatability within each instrument type, the correlation of measurements between instruments, and the agreement of measurements between instruments including bias and precision of the GEM H100 and SE. The Pearson correlation coefficient (r) was calculated to assess the linear correlation of measurements for repeatability within and reproducibility between instruments. The agreement of paired measurements was estimated and plotted using the Bland–Altman method [11]. The Bland–Altman method plots the differences between measurements (vertical axis) against the mean of the two measurements (horizontal axis). The overall average between the paired measurements is plotted with the corresponding 95% confidence interval of agreement limits to summarize the overall average and range of agreement for GEM H100 measurements compared to the SE. We report the percent (%) difference as a metric of bias owing to the increasing variability in measurements as the magnitude of the measurement increased beyond 200 s on the horizontal axis. Passing–Bablok regression analysis was also performed to assess equality between instruments by testing that the intercept (A) and slope (B) equals 0 and 1, respectively. The corresponding 95% confidence intervals associated with each estimate are provided. Statistical significance was defined as an alpha of 0.05, with two-sided alternative hypotheses.

## Results

The SE reference instrument median (IQR) for ACT+ and ACT-LR were, respectively, 444 (127.8, 529) and 167 (148.5, 276.6), with 71% and 29% of measurements greater than 250 s. The GEM H100 average ACT+ and ACT-LR were, respectively, 444 (130, 542.3) and 159 (148.5, 276.5), with 70% and 26% of measurements more than 250 s. For ACT+ , 59% of SE and 60% of GEM H100 measurements were more than 400 s. The majority of ACT+ tests were performed in the cardiovascular operating suite during CPB (Table 1).

**Fig. 1:**
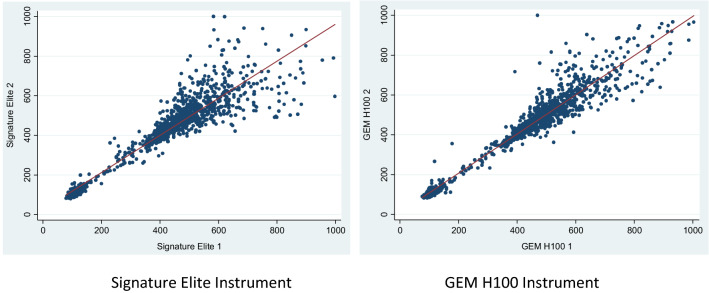
Repeatability of ACT+ (s) measurements by Signature Elite and GEM H100

**Fig. 2:**
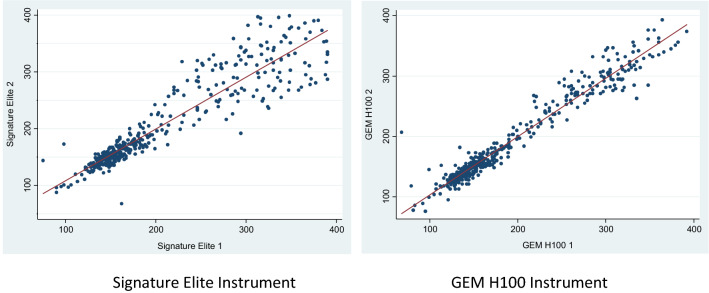
Repeatability of ACT-LR measurements (s) by Signature Elite and GEM H100

There was strong repeatability for ACT+ (Fig. 1) and ACT-LR (Fig. 2) measurements by the SE and GEM H100. Repeatability by the GEM H100 was higher than the repeatability by SE for both ACT+ [rGEM = 0.968 (p < 0.001) versus rSE = 0.943 (p < 0.001) (Fig. 1)] and ACT-LR [rGEM = 0.974 (p < 0.001) versus rSE = 0.9283 (p < 0.001) (Fig. 2)] levels. The higher repeatability of the GEM H100 compared to the SE persisted at ACT levels more than 250 s; respectively, ACT+ [rGEM = 0.878 (p < 0.001) and rSE = 0.761 (p < 0.001)] and ACT-LR levels [rGEM = 0.822 (p < 0.001) versus rSE = 0.338 (p < 0.001)]. The higher repeatability of GEM H100 ACT+ also persisted at ACT levels more than 400 s: rGEM = 0.831 (p < 0.001) versus rSE = 0.652 (p < 0.001).

**Fig. 3:**
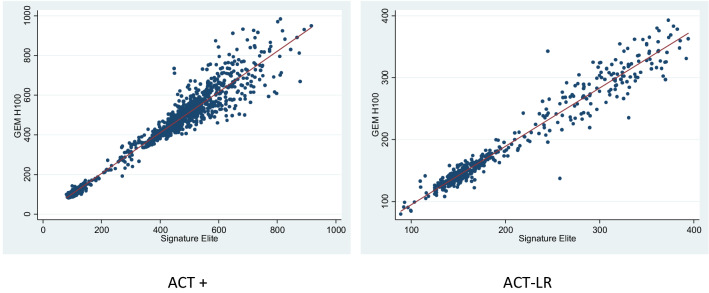
Linear correlation of ACT+ and ACT-LR measurements (s) between instruments

Scatter plots (Fig. 3) comparing the ACT+ measurements between the SE (Fig. 3a) and GH100 (Fig. 3b) indicate a strong linear relationship. Overall, the linear correlation between the two instruments were identical for both ACT+ (r = 0.975; p < 0.001) and ACT-LR (r = 0.975; p < 0.001). The linear relationship between GEM H100 and SE for ACT+ and ACT-LR was strongest when ACT levels were 250 s or less; respectively, r = 0.934 (p < 0.001) and r = 0.929 (p < 0.001). The agreement was reduced when the ACT levels were more than 250 s; ACT+ (r = 0.892; p < 0.001) and ACT-LR (r = 0.789; p < 0.001).

**Fig. 4:**
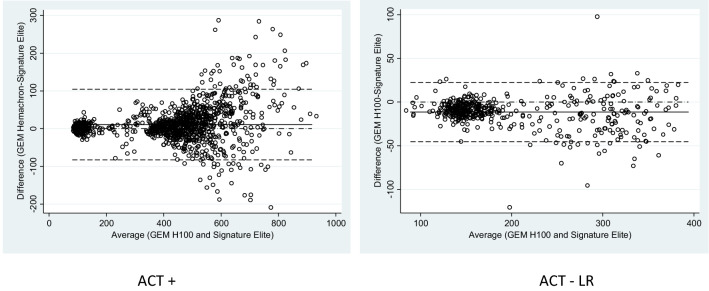
Bland–Altman plots comparing the results of ACT+ and ACT-LR (s) between Signature Elite and GEM H100. The dotted lines represent the 95% limits of agreeement between the two instruments

The Bland–Altman plots (Fig. 4) illustrate a consistent positive bias of the GEM H100 compared to the SE for ACT+ but a negative bias for ACT-LR. On average, ACT measurement differences by GEM H100 were higher than the SE for ACT+ (10.7 ± 47.8) and lower than the SE for ACT-LR (− 11.3 ± 17.2). For both ACT+ (Fig. 4a) and ACT-LR (Fig. 4b), there was increasing variability in the differences of GEM H100 compared to SE as the magnitude of measurements increased. The variability in differences increased significantly when ACT levels were greater than 250 s. When restricted to ACT levels less than 250 s, the bias in the GEM H100 ACT+ measurements were reduced 1.77 ± 9.37 but there was little change in the bias of measurements with the GEM H100 ACT-LR (− 8.68 ± 11.30). The bias of GEM H100 at ACT levels of 250 s or more was 14.46 ± 56.22 for ACT+ and − 17.67 ± 25.66 for ACT-LR. There was small increase in bias for GEM H100 ACT+ at levels more than 400 s: 15.60 ± 60.39.

**Fig. 5:**
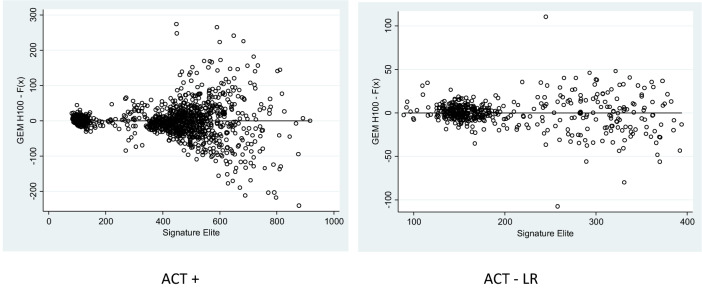
Passing–Bablok residuals for ACT+ and ACT-LR (s)

Figure 5 illustrates the same consistent positive bias of the GEM Hemochron 100 compared to the SE for ACT+ in but a small negative bias for ACT-LR expressed in percentage.

Analysis of the parameters [intercept (A) and slope (B)] of the Passing–Bablok regression demonstrated there was consistent bias of ACT measurements by the GEM Hemochron 100 for both ACT+ (Table 2) and ACT-LR (Table 3). The systematic bias for higher measurements was strongest for ACT+ measurements (Fig. 3a), likely due to most measurements occurring in the CVOR patients, who receive large boluses of UFH. In patients undergoing an EP procedure, the GEM Hemochron 100 performed comparably to the SE.

**Table 2:** Passing–Bablock regression results ACT+ (reference instrument: Signature Elite)

	n	ACT in seconds [median (IQR)]	A	95% CI	B	95% CI
ACT+	1236		5.20	2.74, 7.48	0.96	0.95, 0.97
CVOR	1088	461 (120, 540)	5.06	2.59, 7.77	0.95	0.94, 0.96
EP	147	353 (233, 379)	1.54	−5.80, 8.43	0.99	0.971, 1.018

*CVOR* cardiovascular operating room; *EP* electrophysiology

**Table 3:** Passing–Bablock regression results ACT-LR

	n	ACT in seconds [median (IQR)]	A	95% CI	B	95% CI
ACT-LR	463		−4.16	−8, −0.44	0.97	0.94, 0.99
CCL	204	260.8 (153.8, 318.3)	−4.38	−10.89, 0.67	0.96	0.93, 1.00
CVOR	147	151 (140.5, 167)	4.21	−4.07, 12.07	0.91	0.86, 0.96
ECMO	73	166.5 (152, 180)	2.03	−15.99, 20.04	0.95	0.84, 1.05
EP	23	300 (183.5, 337)	−20.42	−54.79, 14.00	1.06	0.89, 1.19
ICU	16	160.8 (152.5, 166)	−58.44	−204.38, 51.06	1.32	0.63, 2.25

*IQR* interquartile range; *CCL* Cardiac Cath Lab; *CVOR* cardiovascular operating room; *ECMO* extracorporeal membrane oxygenation; *EP* electrophysiology; *ICU* intensive care unit

There was a small but consistent bias for the ACT-LR measurement by GEM Hemochron 100 as the intercept (A) [−4.16 (95% CI −8, −0.44)] did not include 0 (Table 3); however, the slope (B) [0.97 (95% CI 0.94, 0.99) being close to 1 indicates the bias was not proportional. This is likely due to the increasing variability when ACT was more than 250 s. The bias of GEM Hemochron 100 for ACT+ (Table 2) and ACT-LR (Table 3) was greatest in the setting of the CVOR where ACT levels were high. ACT-LR measurements by the GEM Hemochron 100 were comparable to the SE when performed in settings of CCL, ECM, EP, and ICU (Table 2).

## Discussion

This multi-center evaluation of the next generation Hemochron, GEM Hemochron 100, in a variety of clinical situations where unfractionated UFH was administered found the GEM Hemochron 100 to have more consistent clinical performance as compared to the current clinical model Signature Elite.

The correlation between GEM Hemochron 100 devices was especially close at clinically significant values of 100–700 s (Fig. 3). Performance levels with ACT values transiently closer to 1000 s was less precise (Figs. 3a, 4a). The clinical impact of these briefly elevated values is negligible because they only occurred during CPB when large doses of UFH are given and varying responses to UFH may cause these very high values transiently. Patients who were on continuous infusions of UFH at doses used for therapeutic anticoagulation for ECLS had an average ACT level of 164.7 ± 33.4 (Table 2) and, in general, are maintained between 160 and 220 s. Patients receiving UFH for PCI procedures are often monitored with goal ACTs less than 400 [12]. The optimal ACT range for many conditions is still a matter of debate and controversy among clinicians [13].

As compared to the predicate SE, the GEM Hemochron 100 repeatability performance which will be noticeable at the bedside in the clinically relevant ranges of ACT, was superior for both the ACT+ (Fig. 1a) and ACT-LR (Fig. 2a) cartridges. Since it is apparent that the ACT+ cartridge provided comparatively the same repeatability information to the ACT-LR cartridge throughout the measurement range, the need to use 2 different cartridges in one clinical arena may seem unnecessary and cumbersome in some clinical situations. The use of the same cartridge type in a particular clinical setting may be clinically relevant, as needing only 1 type of cartridge may provide savings in inventory management, staff training and in ease of use. Although Kaolin- and Celite-guided management of anticoagulation is clinically not different, the methods are not interchangeable [14]. Given this, using only one cartridge type (ACT+ or ACT-LR) is feasible if establishment of normal and treatable values in any institution is carried out and cartridge types are not used interchangeably.

Of note, patients in the cardiac catheterization suite often did not have baseline ACT’s performed and some patients had no ACT performed at the end of the procedure. Such information may be helpful in determination of the underlying coagulation status of the patient, which may guide initial UFH dosing as well as need for reversal of UFH effect with protamine at the conclusion of the case. Whether following ACT values in both the pre and post procedure time period would affect risk of bleeding is unknown but is a subject for further study.

### Study limitations

This study was limited to clinical settings with adult patients and did not include a pediatric population. It is known that clotting parameters in subjects under the age of 1 year have not fully matured, therefore the measurement of ACT in this population may provide differing results from this adult study. The study also did not test the use of ACT+ only as single cartridge type for all settings, as opposed to the use of ACT-LR and ACT+ . Lastly, we did not attempt to include other equipment manufacturer’s ACT instruments as this would have required the use of more blood and the likelihood of signed informed consenting, which would had significantly impacted enrolment (Table 4).

**Table 4:** Activated Clotting Time Study Procedures

AVR aortic valve replacement
Coronary artery bypass graft surgery
Minimally invasive mitral valve replacement
Electrophysiology procedure
Aortic valve replacement—coronary artery bypass graft
Aortic valve replacement and mitral valve replacement
Minimally invasive aortic valve replacement
Mitral valve replacement
Abdominal aortic aneurysm—aortic valve repair
Ascending aortic aneurysm
Ventricular septal defect repair
Tricuspid valve replacement
Aortic valve replacement—mitral valve replacement-patent foramen ovalle
Aorta repair
Atrial septal defect repair
Mitral valve replacement patent foramen ovale repair
Aortic valve replacement mitral valve replacement-patent foramen ovalle
Heart transplant
Aortic valve replacement—atrial septal defect
Ventricular assist device
Femoral popliteal artery bypass
Transcatheter aortic valve replacement
Extracorporeal life support
Left heart catheterization
Percutaneous coronary intervention
Peripheral intervention
Recurrent slow ventricular tachycardia
nobelstitch
Aortic dissection
Mitral valve replacement—tricuspid valve replacement repair
Mitralclip
Watchmen

## Conclusion

Results obtained for both ACT-LR and ACT+ in all clinical settings in this study using the GEM Hemochron 100 are as accurate and more repeatable as those with the current clinically available Signature Elite. This increase in repeatability between instruments with simultaneously drawn and tested blood samples may answer some of the negative observations associated with the Signature Elite technology. The new technology associated with the GEM Hemochron 100 also offers a future POC coagulation platform, which may benefit clinicians and patient care in the POC setting with menu expansion.
